# Mating-Type Genes Play an Important Role in Fruiting Body Development in *Morchella sextelata*

**DOI:** 10.3390/jof8060564

**Published:** 2022-05-25

**Authors:** Qizheng Liu, Shan Qu, Guoqiang He, Jinkang Wei, Caihong Dong

**Affiliations:** 1 State Key Laboratory of Mycology, Institute of Microbiology, Chinese Academy of Sciences, Beijing 100101, China; liuqz@im.ac.cn (Q.L.); qvshan77@163.com (S.Q.); 2 University of Chinese Academy of Sciences, Beijing 100049, China; 3 Beijing Agricultural Technology Extension Station, Beijing 100029, China; heguoqiang1984@126.com (G.H.); flyinggod99@aliyun.com (J.W.)

**Keywords:** *Morchella sextelata*, mating-type genes, fruiting body, ascus

## Abstract

True morels (*Morchella* spp.) are edible mushrooms that are commercially important worldwide due to their rich nutrition and unique appearance. In recent years, outdoor cultivation has been achieved and expanded on a large scale in China. However, the mechanisms of fruiting body development in morels are poorly understood. In this study, the role of mating-type genes in fruiting body development was researched. Fruiting bodies cultivated with different mating-type strains showed no difference in appearance, but the ascus and ascospores were slightly malformed in fruiting bodies obtained from the *MAT1-1* strains. The transcript levels of mating-type genes and their target genes revealed that the regulatory mechanisms were conserved in ascomycetes fungi. The silencing of *mat1-2-1* by RNA interference verified the direct regulatory effect of *mat1-2-1* on its target genes at the asexual stage. When cultivated with the spawn of single mating-type strains of *MAT1-1* or *MAT1-2*, only one corresponding mating-type gene was detected in the mycelial and conidial samples, but both *mat1-1-1* and *mat1-2-1* were detected in the samples of primordium, pileus, and stipe. An understanding of the mating-type genes’ role in fruiting body development in *M. sextelata* may help to understand the life cycle and facilitate artificial cultivation.

## 1. Introduction

True morels (*Morchella* spp.) are commercially important edible and medicinal mushrooms with a unique appearance and delicate taste [1], belonging to Ascomycota, Pezizomycetes, Pezizales, Morchellaceae, and *Morchella* [2]. In recent years, outdoor cultivation has succeeded and expanded on a large scale in China. However, there are many unsolved basic biological problems, resulting in unstable yields and a high risk in production [3].

In Ascomycetes, mating type is specified by the idiomorph, which carries the sequence of mating-type genes containing two different mating-type loci. Heterothallic fungi require two partners of opposite mating types with compatible *MAT* idiomorphs. In the homothallic species, a single haploid nucleus carries all the information necessary to form fruiting bodies [4]. Mating-type genes are involved in regulating the recognition between different mating types of cells, cell fusion, meiosis processes, and the production of sexual spores, which require appropriate environmental stimulation and signal recognition between strains and are completed by multiple functional molecules [5,6].

Previous research has shown that mating-type genes are expressed throughout the asexual development of *Neurospora crassa* [7]. Studies of single or double knockout of *mat1-1-1* and *mat1-2-1* in *Ulocladium botrytis* showed their importance in colony growth, conidial size, and number [8]. Deletion mutants of mating-type genes were entirely sterile or able to develop stipes but did not form an apothecial disk in *Botrytis cinerea* [9]. Mating-type gene knockout was also reported to lead to the formation of sterile apothecial disks in *Sclerotinia sclerotiorum* [10]. Recent evidence suggests that mating-type genes play an important role in asexual and sexual reproduction.

By sequencing the genomes of monospore strains with opposite polarity, it was found that there were two mating types in *Morchella importuna* [11]. Moreover, there were genetic differences among the strains of the *MAT1-1* and *MAT1-2* mating types, and the proportions of endemic genes were 5.79% and 7.37%, respectively, suggesting that *M. importuna* was a heterothallic fungus [11]. It has previously been observed that the idiomorph contains *mat1-1-1* and two further newly described *MAT* genes, *mat1-1-10* and *mat1-1-11*, from analysis of the sequence of *MAT1-1* idiomorph in *M. importuna* [12]. In the Elata clade which contains *Morchella sextelata*, both *mat1-1-10* and *mat1-1-11* were found [13]. Data from a total of 186 black morel ascocarps showed that most of the pileus in the wild fruiting bodies were the double mating type, most of the stipes were the single mating type, and the proportion of *mat1-1-1* in the stipe was higher than that of *mat1-2-1*, while the stipe and pileus of the fruiting bodies obtained from outdoor cultivation were mainly the double mating type [14].

The process of the artificial cultivation of *Morchella* is affected by various environmental and biological factors, such as the biodiversity of fungi and bacteria in fruiting soils [15,16], the time of the addition of exogenous nutrients [17], etc. Most studies in the field of mating-type genes of morel have only focused on gene identification and the characteristics of the distribution. However, very little is known about the role of mating-type genes in the fruiting body development of *Morchella*. The main challenge faced by many researchers is the difficulty of artificial cultivation. The purpose of this investigation was to explore the relationship between mating-type genes and fruiting body development. The present research revealed, for the first time, the functions of mating-type genes in *M.* *sextelata* and found that mating-type genes regulated ascus and ascospore development.

## 2. Materials and Methods

### 2.1. Strains and Culture Conditions

The *M. sextelata* strain was deposited in the China General Microbiological Culture Collection Center (CGMCC No. 3.20953). The ascospores were collected from a fruiting body by cultivation of strain CGMCC No. 3.20953 in the Luquan District, Shijiazhuang City, Hebei Province, China. The single-ascospore strains were isolated by gradient dilution and spreading the ascospores on an agar-only medium. The germinated strains were identified as *M. sextelata* by blasting the ITS-rDNA sequence at the *Morchella* multilocus sequence typing (*MLST*) website (https://wi.knaw.nl/page/Pairwise_alignment, accessed on 18 March 2019) [18]. The mating-type was determined by *MAT* locus sequence amplification [12]. The strains M1A and M1B were *MAT1-1* type; the strains M2A and M2B were *MAT1-2* type strains.

The strains were routinely grown at 20 °C on a potato dextrose agar (PDA) medium (200 g of potatoes were boiled in 1000 mL distilled water; then, 20 g of dextrose and 18 g of agar were added to 1000 mL of potato extract water) in the dark. Luria–Bertani (LB) medium containing 50 μg/mL of kanamycin was used to incubate *Escherichia coli* DH5α for plasmid amplification. *Agrobacterium tumefaciens* strain EHA105 (Cat No. AC1010S, Weidi Biotechnology Co., Ltd., Shanghai, China) was used for fungal transformation. The media used for Agrobacterium-mediated transformation, including a complete yeast medium (CYM), a minimal medium (MM), an induction medium (IM), and a cocultivation medium (CoM), were as previously presented [19].

### 2.2. Fruiting Body Cultivation and Sample Collection

The strains were cultured on PDA plates for 7 days, and mycelial samples were collected. The strains with the *MAT1-1* type, *MAT1-2* type, and a mixture of *MAT1-1* and *MAT1-2* type were cultivated in the Changping District, Beijing, China (N: 40°08′35.61″ E: 116°20′45.62″). Sowing and post-sowing were carried out according to the previous report [3]. Common vegetable garden soil was used for cultivation, and the soil humidity was maintained at 50–70%. The soil treatment was placed into approximately 1.5 square meters of quadrats. Spawning began when the highest local temperature was below 20 ℃ in November. We crushed the spawns and covered them with soil after trench sowing 500 g of spawns per quadrat. The conidial samples were collected from the soil surface 10 days after spawning. Exogenous nutrition bags (ENB) were added after the conidia were spread on the soil surface. When the temperature increased to 8–10 ℃ in the spring, drip irrigation was performed for water replenishment and maintaining the air humidity of 85–90% and the soil moisture of 65–75%. These conditions will stimulate the differentiation of the primordium of the *M. sextelata*. The primordial samples were collected when the primordia formed. The temperature of the greenhouse was kept at about 20 ℃ to provide the optimum temperature for fruiting body development. The fruiting body samples were tissue blocks cut from the pileus and stipes of mature fruiting bodies over 8 cm in length.

The primordia appeared after 2 months of spawning and were harvested after 1 month. The media for spawn and ENB were as follows: spawn—wheat 46%, husk 20%, wheat bran 18%, sawdust 10%, gypsum 1%, precipitated calcium carbonate (PCC) 1%, and humus 4%; ENB—wheat 67%, sawdust 28%, and lime 5%.

### 2.3. Morphological Observation and Measurement

Mature fruiting bodies of more than 8 cm were selected for morphological observation, and the pileus was crosscut with a scalpel to obtain slices with a thickness of approximately 1 mm and observed under a dissecting microscope (SMZ1500, Nikon, Minato-ku, Tokyo, Japan). Ascus and ascospore observations were performed with a Nikon Eclipse 80i microscope (Nikon, Minato-ku, Tokyo, Japan). To visualize the nuclei, ascospores were stained with 4′-6-diamidino-2-phenilindole (DAPI). The ascospores were incubated directly in the staining reagent on the microscope slide for 15 min [20]. The thickness of each layer of pileus in the fruiting bodies and the size of ascus and ascospore were measured using ImageJ software (http://rsb.info.nih.gov/ij, accessed on 10 May 2020).

### 2.4. Construction of RNAi Plasmids for mat1-2-1

The dual promoter RNAi plasmid pFGL815-35S-gpd was constructed as described in a previous study [21]. Briefly, a 660-bp promoter sequence of *gpd* and a 1200-bp 35S promoter were amplified from *M. sextelata* genomic DNA and pFGL815, respectively. Two sequences were conjugated by fusion PCR and then inserted into the *Hind*III and *Spe*I sites of the plasmid pFGL815-hyg using a T4 DNA ligase (NEB, Boston, MA, USA). Then, a 440-bp functional sequence of *mat1-2-1* was inserted using a ClonExpress Ultra One Step Cloning Kit (Cat No. C115, Vazyme Biotech Co., Ltd., Nanjing, China). Finally, RNA silencing plasmids were obtained and named pFGL815-hyg-ms-mat121-RNAi. All related primers are listed in Table 1.

### 2.5. Agrobacterium-Mediated Transformation of M. sextelata

Transformation was performed according to the procedure used by Lv et al. [19]. Briefly, strains pregrown in the dark at 20 °C for 5 days on CYM were used as transformation materials. For screening the transformants, hygromycin (hyg) at a concentration of 10 µg/mL was used. LB medium was used to cultivate *A. tumefaciens* harboring the pFGL815-hyg-ms-mat121-RNAi vector overnight, diluted (1:100) into 100 mL of MM, and incubated overnight at 28 °C again. *Agrobacterium* cells were harvested by centrifugation (5000 rpm for 10 min) and resuspended in 100 mL of IM to a density of 0.4–0.6 (OD600), followed by incubation (28 °C, 100 rpm) for 4–6 h. Mycelial plugs from the CYM plate were immersed in the culture of preinduced *A. tumefaciens* for 30 min and placed on CoIM for cocultivation (28 °C, 2 days). Then, the mycelial plugs were transferred to the selection medium (300 µg/mL cefotaxime and 10 µg/mL hyg). The germinated plugs were also transferred to the selection medium.

### 2.6. DNA Extraction and PCR

Genomic DNA was extracted from mycelia and tissue blocks using the cetyltrimethylammonium bromide (CTAB) method [22]. Samples were crushed to powder in liquid nitrogen, 500 µL of CTAB was added, and the mixture was placed in a 65 °C water bath for 30 min. After extraction with the phenol/chloroform/isoamylol, the DNA was dissolved with 50 µL of ddH_2_O.

The primers used for PCR analysis of the *hyg*, *mat*, and target genes are shown in Table 1. PCR was performed with a 2× mix (Cat No. P222-w1, Vazyme Biotech Co., Ltd., Nanjing, China), and the following procedures were used: denaturation at 95 °C for 3 min, 35 amplification cycles (denaturation at 95 °C for 30 s, annealing at 55 °C for 30 s, and elongation at 72 °C for 15 s), followed by a final elongation step at 72 °C for 7 min.

### 2.7. RNA Extraction and Reverse-Transcription (RT)-PCR

Fresh mycelia were collected for RNA isolation as described in the previous report [23]. Mycelia and tissue blocks were broken using a Microsmash disrupter (Tomy Medico, Nerima-ku, Tokyo, Japan) at 4 °C. TRIzol reagent (Invitrogen, Carlsbad, CA, USA) was used to extract total RNA. Extracted RNA was then treated with RQ1 RNase-Free DNase (Promega, Madison, WI, USA).

Gene sequences were searched from JGI (https://genome.jgi.doe.gov/Morco1/Morco1.home.html, accessed on 20 July 2020). The oligonucleotide primers used were listed in Table 1, and primers for *act1* were performed as previously reported [23]. The intensity of each band in the gel images was measured using ImageJ software, and the ratio of tested gene expression to *act1* expression was calculated.

## 3. Results

### 3.1. Mating Type Detection at the Different Stages

**Figure 1:**
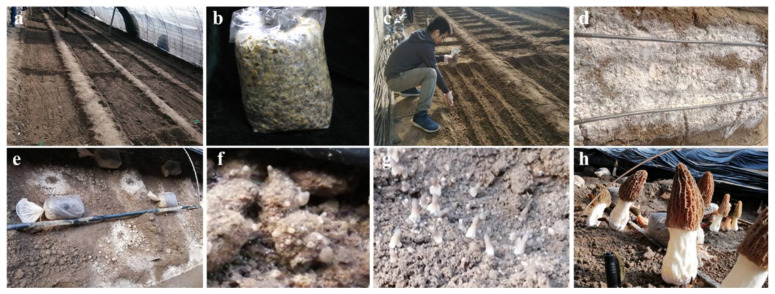
Fruiting body cultivation of morel. (**a**) Prepared soil; (**b**) spawn; (**c**) sowing the spawn; (**d**) conidia (‘powdery mildew’); (**e**) adding exogenous nutrition bags (ENB); (**f**) primordia; (**g**) delineating pileus and stipe; and (**h**) mature fruiting body.

**Figure 2:**
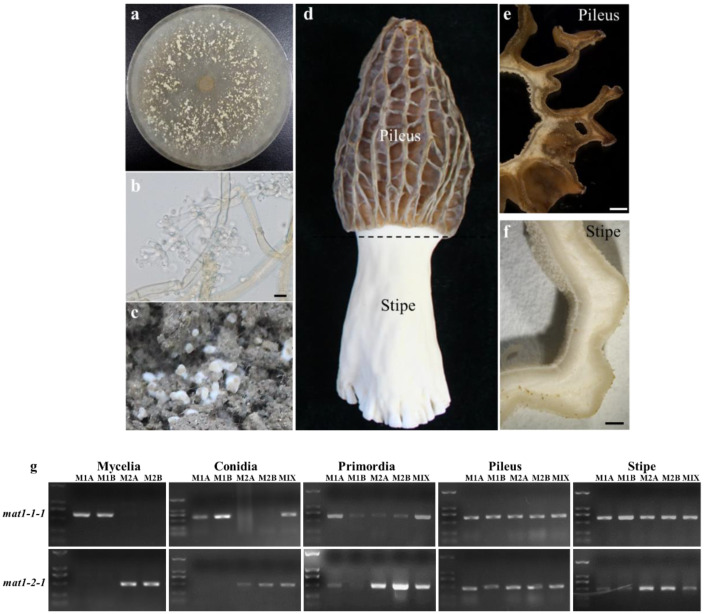
The sample collection and gene detection at different stages. (**a**) Samples of mycelia; (**b**) conidia, bar = 10 μm; (**c**) primordia; (**d**) fruiting body; (**e**) pileus, bar = 1 mm; and (**f**) stipe, bar = 1 mm. (**g**) The PCR results of mating-type genes of different samples at different stages. M1A and M1B are *MAT1-1* type, M2A and M2B are *MAT1-2* type, and MIX is spawned mixing *MAT1-1* with *MAT1-2* type strains.

Strains of different mating types were cultivated (Figure 1a–h). The samples including mycelia, ‘powdery mildew’, which contained the conidia, primordia, pileus, and stipe of the fruiting body, are shown in Figure 2a–f. By PCR analysis, mating-type gene sequences were determined during the course of cultivation (Figure 2g). Among the samples cultivated with single mating-type strains of *MAT1-1* or *MAT1-2*, only one corresponding mating-type gene was detected in the mycelial and conidial samples, but both *mat1-1-1* and *mat1-2-1* were detected in the samples of the primordium, pileus, and stipe. As expected, two mating-type genes could be detected in samples of all stages of the mixed-seeded quadrat.

### 3.2. Different Mating Types Generate Divergent Physiological Structures of Fruiting Bodies

**Figure 3:**
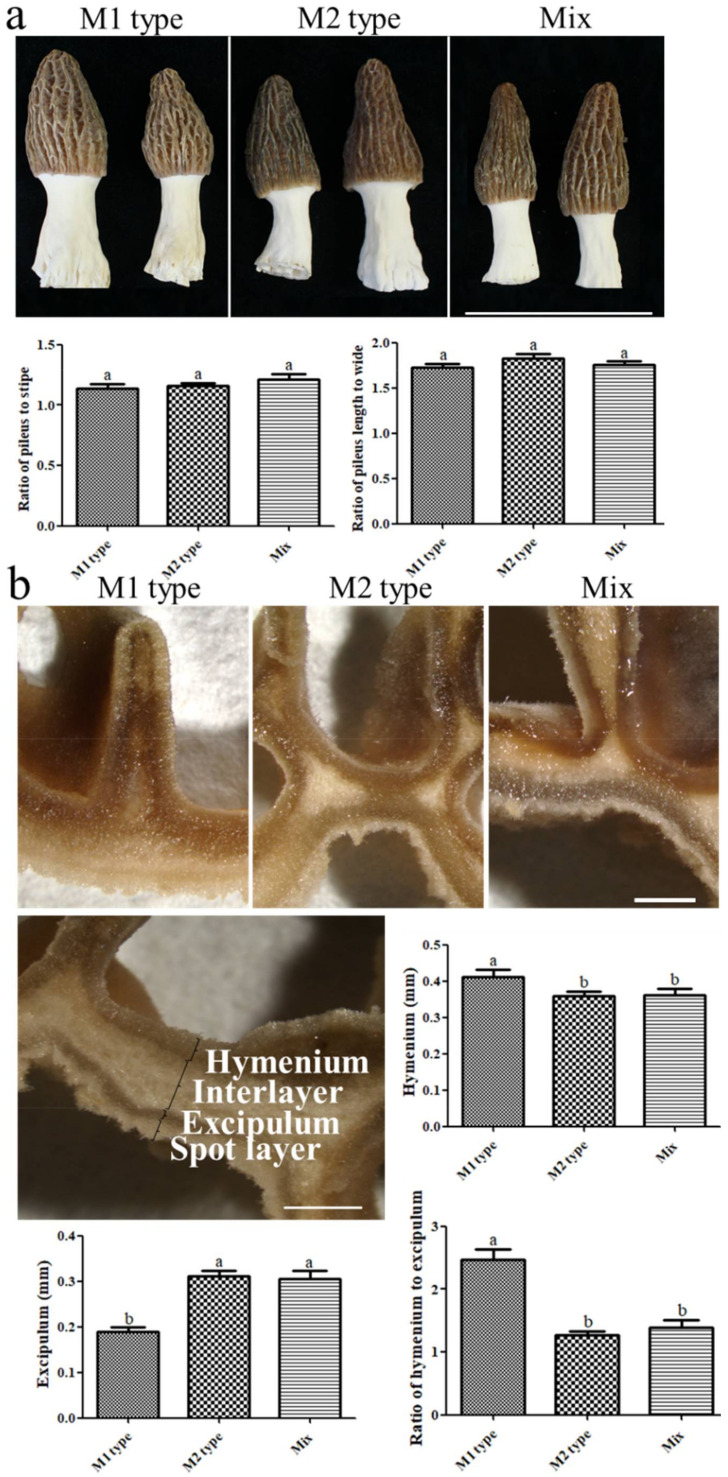
Appearance and structure of the fruiting body cultivated with strains of different types. (**a**) Appearance of different fruiting bodies, bar = 10 cm. (**b**) The structure division, shape and size of each layer of fruiting body, bar = 1 mm. Different letters above the bars indicate significant differences, one-way ANOVA, *p* < 0.05; the ANOVA results are shown in the supplementary files (Table S3.1–3.5).

The fruiting bodies obtained by cultivation with the *MAT1-1* type strain or *MAT1-2* type strain were defined as M1 type and M2 type fruiting bodies, respectively. The fruiting bodies obtained from hybridizing the *MAT1-1* type strain with the *MAT1-2* type strain were defined as the Mix type. No significant differences were found between the three types of fruiting bodies in the length ratio of pileus to stipe and the size ratio of pileus length to width (Figure 3a). There was no difference in the macroscopic morphology of the different fruiting bodies.

From the cross-cutting of the pileus, four layers could be separated, and we defined these as the hymenium layer, the interlayer, the excipulum layer, and the spot layer (Figure 3b). The thickness of the hymenium of the M1 type (n = 65) was significantly greater (*p* < 0.05) than that of the M2 type (n = 75) and the Mix type (n = 50). However, the thickness of the excipulum in the M1 type was significantly less (*p* < 0.05) than that of the other two types. In particular, the thickness ratio of the hymenium to the excipulum in the M1 type was larger than that in the other two types (Figure 3b). As shown in Figure S1, there was a layer of tissue inside the pileus, and the surface was full of spot structures. According to the statistical analysis of the thickness of the spot layer, the M1 type was thinner than the M2 type, and the Mix type was the thickest. The thickness of the interlayer of the M1 type was less than that of the Mix type, while the M2 type was not significantly different from the other two types (Figure S1). Both the thickness of the spot layer and the interlayer showed the same trend: the M1 type was less than the other two types. In general, there was a large difference in the organizations of the strains with different *MAT* types.

### 3.3. Mating Type Influences Ascus and Ascospore Development

**Figure 4:**
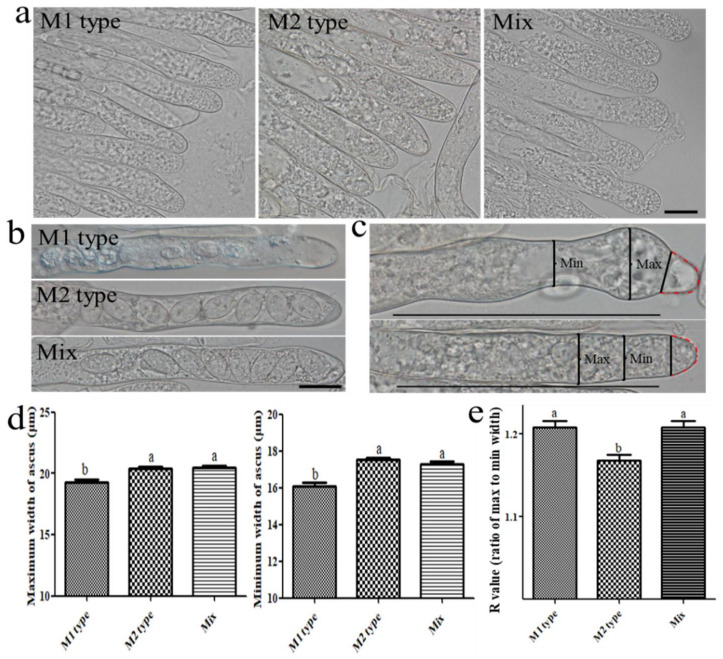
Morphology of the ascus of different mating types. (**a**,**b**) Morphology of the ascus of different types of fruiting bodies, bar = 20 μm; (**c**) the malformation degree measurement instructions, bar = 100 μm; (**d**) maximum and minimum size of the ascus; (**e**) the malformation degree of the ascus of different types. Different letters indicate significant differences (Table S3.6–3.8), one-way ANOVA, *p* < 0.05.

The influences of different mating types on ascus development were examined under a microscope. The morphology of the immature ascus and mature ascus is shown in Figure 4a,b. It can be seen that the ascus of the M1 type was somewhat abnormal compared with the other two types. Observation of the ascus was performed according to the following criteria: the maximum and minimum widths within 100 μm of the front end were measured ignoring the anterior arcuate region (delineated by the red dotted line in Figure 4c). A parameter R was set to describe the malformation degree of the ascus, and the R value was obtained by calculating the max width/min width (ratio). The greater the difference between the maximum and the minimum width, the greater the R value, which indicated the higher the malformation degree of the ascus. The maximum and minimum width data of the ascus (Figure 4d) showed that the size of the M1 type ascus (n = 170) was smaller than that of the other two types, and there was no significant difference between the M2 type (n = 182) and Mix type (n = 300). The results of the R value showed that the M2 type was significantly smaller than the other two types, and the M1 type and Mix type had no significant difference (Figure 4e). Overall, these results indicated that the malformation degree of the M1 type ascus was higher than that of the M2 type ascus, and the size was smaller than that of the M2 type ascus. The malformation degree of the Mix type ascus was the same as M1 type, but the size was the same as the M2 type.

**Figure 5:**
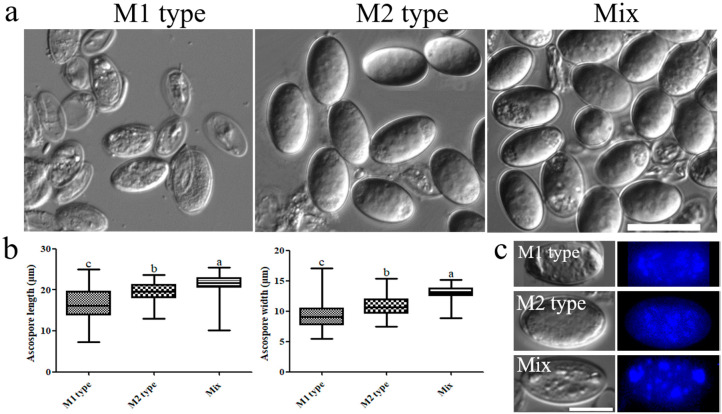
Morphology analysis of the ascospore in different types of fruiting bodies. (**a**) Morphology of the ascospores from different types of fruiting bodies, bar = 20 μm; (**b**) the size of the ascospores. Different letters indicate significant differences (Table S3.9 and 3.10), one-way ANOVA, *p* < 0.05; (**c**) photograph of 4’,6-diamidino-2-phenylindole (DAPI) staining of ascospores, bar = 10 μm.

The morphology of the ascospores obtained from the three types of fruiting bodies was also observed under a microscope (Figure 5a). The measurement of the length and width of the ascospores indicated that the size of the M1 type (n = 70) was significantly smaller than that of the M2 type (n = 70), and the size of the Mix type (n = 70) was the largest (Figure 5b). Nuclear staining by DAPI showed that there were multiple nuclei, consistent with the results of Du et al. [20], and there was no difference in the number of nuclei between the three types of ascospores (Figure 5c).

### 3.4. Transcription Levels of Mating-Type Genes and Ascus Development-Related Genes in Pileus

**Figure 6:**
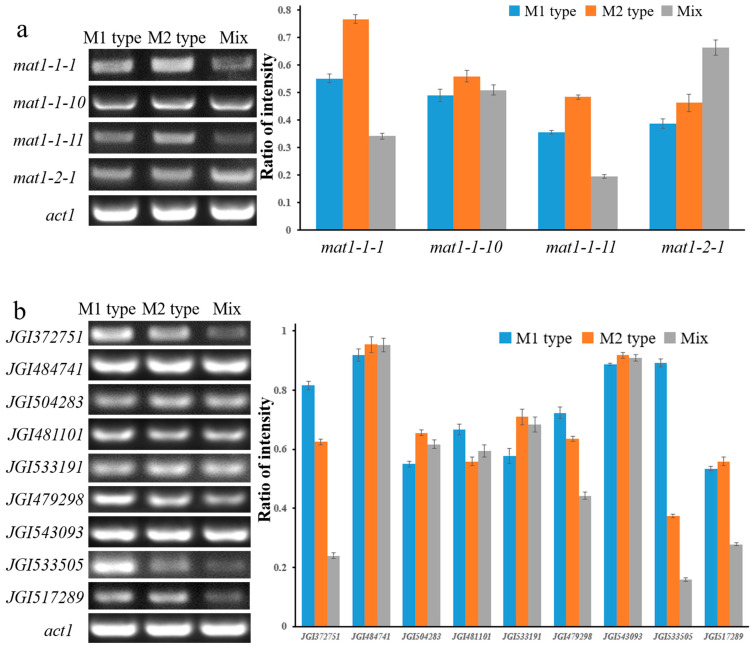
The transcription level of mating-type genes and ascus development-related genes in pileus. (**a**) Expression of mating-type genes detected by reverse-transcription (RT)-PCR in different tissue blocks (Table S4.1). The intensity ratio indicated mating-type gene expression versus *act1* expression. (**b**) Reverse transcription (RT)-PCR was performed to detect the expression of genes related to ascus development in different tissue blocks (Table S4.2). Constitutive *act1* served as the control. The intensity ratio indicated target gene expression versus *act1* expression. Error bars indicate standard deviation.

RT-PCR was used to detect the transcription levels of each mating-type gene in different types of pilei (Figure 6a). The results showed that the transcription levels of *mat1-1-1*, *mat1-1-10*, and *mat1-1-11* were the highest in the M2 type and the lowest in the Mix type. There was little difference in the transcription level of *mat1-1-10* in all types of pilei. The transcription levels of *mat1-1-1* and *mat1-1-11* in the Mix type were lower than those in the M1 type and M2 type. The transcription level of *mat1-2-1* was the highest in the Mix type and the lowest in the M1 type. In general, the transcription level of each gene in the M1 type was slightly lower than that of the M2 type, and the Mix type pileus was significantly different from that of the M1 type and the M2 type.

Previously, it was reported that some ascus development-related genes were regulated by mating-type genes in *Fusarium graminearum* [24], and several homologous sequences (Table S1) were found by comparison with the *M. sextelata* genome (GenBank assembly accession: GCA_009741755.1). However, the lack of annotation information led us to compare the homologous sequences with the *M. importuna* genome in JGI [25] (https://mycocosm.jgi.doe.gov/Morco1/Morco1.home.html, accessed on 20 July 2020). The results showed that they were almost the same in gene sequences (Table S2); so, we named these homologous sequences with protein IDs from the JGI database. The transcription levels of these target genes were detected by RT–PCR, with *act1* as the reference gene. It has been reported that FGSG_00404, FGSG_09896, and FGSG_09834 were inhibited by *mat1-2-1* [24], and JGI372751, JGI479298, and JGI533505 were homologous genes in *M. sextelata*, respectively. The results showed that the three genes had the lowest expression level in the Mix type pileus, while the expression level was relatively high in the M1 type pileus (Figure 6b), which was in line with expectations. In addition to the low expression level of JGI517289 in the Mix type, no significant differences were detected in the other genes (JGI484741, JGI504283, JGI484101, JGI533191, and JGI543093), as shown in Figure 6b.

### 3.5. mat1-2-1 Regulated Ascus Development-Related Genes at the Mycelial Stage

**Figure 7:**
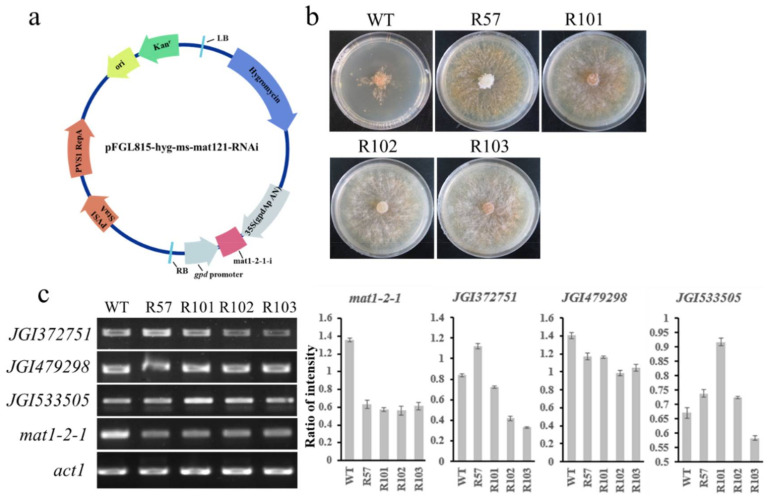
Phenotype of the *MAT1-2* strain after the silencing of *mat1-2-1* by RNA interference. (**a**) The construction of plasmids pFGL815-hyg-ms-mat121-RNAi. (**b**) Wildtype strain and transformants after 7 days of cultivation on PDA supplemented with 10 μg/mL hygromycin B. (**c**) The expression levels of *mat1-2-1* and its target genes in different transformants on PDA. Constitutive *act1* served as the control. Intensity ratio indicates gene expression versus *act1* expression; error bars indicate standard deviation.

RNA interference experiments were performed to verify the regulatory role of *mat1-2-1* at the asexual stage. The constructed vector pFGL815-hyg-ms-mat121-RNAi (Figure 7a) was transformed into the *MAT1-2* strain M2B by the Agrobacterium-mediated transformation method, and four transformants were obtained through multiple rounds of screening. The growth of wildtype and transformants on a 10 μg/mL hygromycin PDA plate is shown in Figure 7b. The PCR detection results of *hyg* in the transformants are shown in Figure S2. The transcription level of *mat1-2-1* in transformants was detected, and the expression level of *mat1-2-1* in transformants decreased significantly (Figure 7c). We also detected the gene expression levels of the three target genes negatively regulated by *mat1-2-1* in the transformants and found that the expression level of JGI533505 was significantly upregulated, while the other two genes were not significantly upregulated (Figure 7c). The results showed that some genes related to ascus development were also expressed at the mycelia stage, and JGI533505 was regulated negatively by *mat1-2-1*, while the other two target genes may have weak negative regulatory effects at this stage.

## 4. Discussion

Mating-type genes have been reported to play an important role in fruiting body development in multiple species [9,10]. The characteristics of the mating-type genes in *Morchella* and the mating system in the life cycle of *Morchella* have been reported recently; however, the function of the mating-type genes is still unknown. In this study, it was found that normal fruiting body development requires both mating types together, and the regulatory mechanism of the target genes is conservative in ascomycetes fungi.

### 4.1. Is It Mating-Type Switching?

The divergent spatial distribution of both mating types in natural morel populations and cultivated sites were monitored [14]. It was found that the fertile tissue of fruiting bodies usually harbored both mating types, whereas sterile tissue of wild morels constantly had one *MAT* allele. *MAT1-1* was detected significantly more commonly than *MAT1-2* in the natural population, suggesting a competitive advantage for *MAT1-1* strains [14]. For the cultivated morel, both the stipes and the pileus always exhibited both *MAT* alleles, which was presumed to be related to the abundance of nutrients in the field soil [14].

To investigate the changes in mating-type genes during cultivation, samples at different stages were analyzed. When strains of the single mating types, *MAT1-1* or *MAT1-2*, were cultivated, only one corresponding mating-type gene was detected in the mycelial and conidial samples, but both *mat1-1-1* and *mat1-2-1* were detected in the samples of primordium, pileus and stipe. The results were confirmed many times. The stipe and pileus of the fruiting bodies were double mating types, which was consistent with previous reports [14]. Monosporic spawns can obtain double mating-type fruiting bodies through cultivation. This phenomenon has been reported and is speculated to be caused by the natural transmission of conidium [26]. However, since no opposite mating-type gene was detected at the conidial stage of the monosporic strains, it was speculated that, in addition to asexual spore transmission, there might be genetic material exchange before or after the conidial stage.

The mechanism of mating-type switching is clear in some yeast species and is controlled by a reversible programmed DNA-rearrangement process [27]. However, it is not clear in some other species, such as *Cordyceps militaris* [28]. Although there was no direct evidence, we still speculate that there may be some unknown mechanism of mating-type switching in *Morchella*. It is necessary to further study this mechanism to better understand the life cycle and fruiting body development mechanism of *Morchella*.

For example, some factors to be considered in the future, include whether different strains may diffuse and mix via soil mycelium expansion and even aerosol diffusion, etc. Perhaps the independent cultivation could be performed by referring to the tray cultivation mode of Tan et al. [29]. Recently, reports have speculated that all ascospores in *M. importuna* are heterokarytic, and the opposite mating type nuclei are asymmetrically distributed in mycelia germinated from single ascospore [30]. Therefore, *M. importuna* is a pseudohomothallism ascomycete fungus [30]. It is noteworthy whether there is a similar phenomenon in *M. sexelata*. More detailed work should be carried out in the future to explain the changes in mating-type genes during the cultivation of *Morchella*.

### 4.2. Mating-Type Genes Regulate Fruiting Body Development

The fruiting bodies obtained by different mating-type strains had no difference in appearance, for example, the ratio of pileus to stipe. It was found that the thickness of the excipulum layer of the M1 type fruiting body was smaller, and the size of the ascus and ascospores were also smaller than that of the other two groups. The M2 type had the lowest degree of ascus deformity. The ascospore size of Mix type was the largest. From the observations, there were differences among the different types of fruiting bodies in structure.

The transcription levels of *mat1-1-1* and *mat1-2-1* in the pileus were the lowest in the M1 type, *mat1-1-1* was the highest in the M2 type, and *mat1-2-1* was the highest in the Mix type. Although both mating-type genes could be detected in the three types of fruiting bodies, their expression levels were significantly different, indicating that the proportion of the two mating-type cells in each fruiting body was different. The reason for the different proportions may be that the number of the two types of cells was different from sowing; on the other hand, it may also be caused by the different growth capacities of the two types of mycelia. Du et al. [14] speculated that *MAT1-1* type cells had a growth advantage; however, this prediction was based on the distribution of *mat1-1-1*, without considering the structure of the fruiting body and mating-type genes expression levels.

Some genes were positively regulated by mating-type genes, while others were negatively regulated [24]. From the perspective of the fruiting body structure, the regulatory mechanisms of those genes on different structural developments were also different. Some may promote the development of the excipulum but inhibit the hymenium in the pileus. However, due to the lack of research, only a small number of morel fruiting body development-related gene expression levels can be detected at present, and these data are not enough to explain the causes of different structures in the three types of fruiting bodies. The fruiting body development-related genes of *Morchella* need to be further studied.

**Figure 8:**
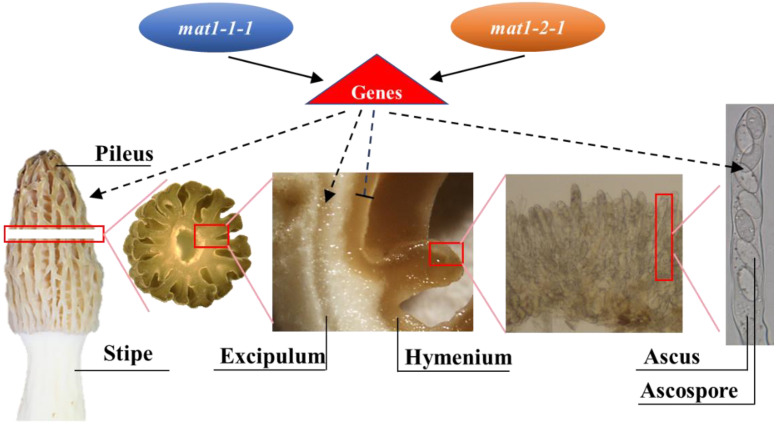
Coregulation model of mating-type genes on fruiting body development in *Morchella*. A group of genes play different roles in the hymenium, excipulum, and ascus development. Mating-type genes, such as *mat1-1-1* or *mat1-2-1*, can regulate these genes. The expression level of each mating-type gene showed an important role in the orderly development of the fruiting body. Dotted arrows represent the predictive impact of genes, the triangle represents promotion, and T represents inhibition. Solid arrows represent the reported relationship in other papers or examined in this paper. The red box indicates that the area was magnified.

The transcription levels of mating-type genes and downstream target genes in the three types of fruiting bodies were different, which resulted in structural differences in fruiting bodies at the macro and micro levels. Fruiting body development is a complex process, and a single mating-type gene cannot completely regulate the development of the fruiting body. Based on these results, we hypothesize that the normal development of the fruiting body requires coregulation by mating-type genes (Figure 8). When the *mat1-1-1* and *mat1-2-1* expression levels were decreased, and the ability to regulate genes was reduced, the function of the genes was weakened. For instance, as shown in the M1 type pileus, the two mating-type gene expression levels were the lowest, leading to a small excipulum and large hymenium size and more deformities of the ascus and ascospores.

### 4.3. A Conservative Mechanism of Controlling Target Gene Expression by Mating-Type Genes

The genes regulated by *mat1-1-1* are involved in sexual development, morphogenesis and asexual development, and amino acid and secondary metabolism in *Penicillium chrysogenum* [31]. By cDNA hybridization, 248 sequences were found to be *mat1-2-1* target genes; 55% of these genes were positively regulated by *mat1-2-1* in *Fusarium verticillioides*, and these target genes were involved in processes such as protein synthesis, metabolism, and cell signaling [32].

We detected the gene expression levels in different types of fruiting bodies and found that the expression trend of genes was consistent with the prediction. The expression level of *mat1-2-1* was the highest in the Mix group, and the expression level of its negatively regulated target genes was the lowest compared with the other two groups, which was consistent with the trend reported for other species [24]. After silencing *mat1-2-1* in the *MAT1-2* strain, the transcription level of some target genes increased, which verified the direct negative regulation of *mat1-2-1* on the target gene. The results showed that the regulatory mechanism of mating-type genes in some ascomycetes fungi is conserved. This study is helpful for understanding the mechanism of morel fruiting body development and provides a theoretical basis for artificial cultivation and strain breeding of *Morchella*.

## Figures and Tables

**Table 1:** Primers used in this study.

Name	Sequence (5′ to 3′)
mat1-1-1-QL	GCCTTCTGAGT CCGTTAT
mat1-1-1-QR	ATGTGAGCGTCCCTTGA
mat1-2-1-QL	GCCGTGACCCTCCTTCT
mat1-2-1-QR	TCGTTGTTCCCAATCCC
hyg-F	CTGTCGAGAAGTTTCTGATCG
hyg-R	CTGATAGAGTTGGTCAAGACC
mat1-1-1-RT-F	TGTCTTCGTAACGCCACT
mat1-1-1-RT-R	TAGCCCAACCCTTCCA
mat1-1-10-RT-F	TCGGAGGATGTTGGGTT
mat1-1-10-RT-R	ACGCTGCTTGAAGTATGGA
mat1-1-11-RT-F	GTGAAGCCATCAACAA
mat1-1-11-RT-R	GAGCCTTTCGTCAATAC
mat1-2-1-RT-F	CCAGGGAAAGAAAGTG
mat1-2-1-RT-R	CATAGGACGAGGAACAT
372751-RT-F	TGGACCGATTAGGGAG
372751-RT-R	TGTTGTTGCGGATGAC
484741-RT-F	ACATTGTCACCACCCTC
484741-RT-R	CCAAACTACTCGCCTTC
504283-RT-F	TAAGGACGGCTATGTAA
504283-RT-R	TCCGCAACTAGACCAC
481101-RT-F	ACCTTACCCAACCTGA
481101-RT-R	CTATCGTCCGCATTTA
533191-RT-F	TCAACTACCACCTCACCC
533191-RT-R	TCCCTCGCCAAAGAAC
479298-RT-F	AACACTCTTGACCTCCAC
479298-RT-R	CATCAGTAACCGCCTC
543093-RT-F	GAACACTTACACGCCTAC
543093-RT-R	CATCTCAGCCATCTCG
533505-RT-F	ATTCCACCAAACAACG
533505-RT-R	CCAACATAAACACCACC
517289-RT-F	TGTCACCTCCATCTGTC
517289-RT-R	GTATTCGGTCCGTCAT
HindIII-GPD-F1	TGCCAAGCTTCGGGAGGTACGGGGAGAATA
SbfI-GPD-B1	CAACCTGCAGGTTTGACTATTTAGTG
SpeI-35S-F3	GAAACTAGTGTTTGATCGAGACCTAATACAGCC
SbfI-35S-B3	ATTCACTAAATAGTCAAACCTGCAGGGGTGATGTCTGCTCAAGC
Part-mat1-2-1-F	TTGAGCAGACATCACCCCTGCAGGCTTCGGCCAGAACAGATG
Part-mat1-2-1-R	ATTCACTAAATAGTCAAACCTGCAGGGCATTCATAGGACGAG

## Data Availability

Not applicable.

## Supplementary Materials

The following supporting information can be downloaded at: https://www.mdpi.com/article/10.3390/jof8060564/s1, Figure S1: The size of spot layer and inter layer in different type of fruiting bodies; Figure S2: PCR check of *hyg* in different transformants and WT strain; Table S1: List of genes that regulated by mating-type genes; Table S2: Blast results of target genes of mating-type genes in *M. sextelata* and *M. importuna*; Table S3.1–10: ANOVA results of List VAR00001:1.00, 2.00 and 3.00 represent M1 type, M2 type and Mix type, respectively; Table S4.1: The transcription level of mating-type genes in pileus; Table S4.2: The transcription level of ascus development-related genes in pileus.
