# Supplementary material for: Mating-Type Genes Play an Important Role in Fruiting Body Development in Morchella sextelata
Source: J Fungi (Basel). 2022 May 25;8(6):564. doi: 10.3390/jof8060564 (PMC9225556; doi:10.3390/jof8060564)
Supplement: Supplementary file 1 [file jof-08-00564-s001.zip › jof-1734966-supplementary.pdf]

Mating-Type Genes Play an Important Role in Fruiting Body Development in *Morchella sextelata*

Supplementary materials

Qizheng Liu [1], Shan Qu [1,2], Guoqiang He [3], Jinkang Wei [3] and Caihong Dong [1,*]

[1] State Key Laboratory of Mycology, Institute of Microbiology, Chinese Academy of Sciences, Beijing 100101, China; liuqz@im.ac.cn (Q.L.); qvshan77@163.com (S.Q.)
[2] University of Chinese Academy of Sciences, Beijing 100049, China
[3] Beijing Agricultural Technology Extension Station, Beijing 100029, China; heguoqiang1984@126.com (G.H.); flyinggod99@aliyun.com (J.W.)
* Correspondence: dongch@im.ac.cn

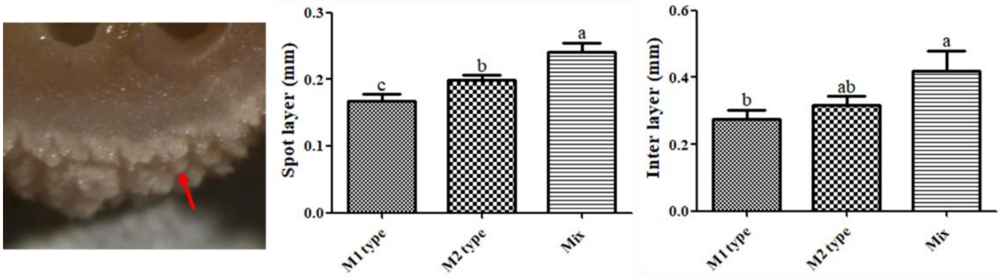

Figure S1. The size of spot layer and inter layer in different type of fruiting bodies.

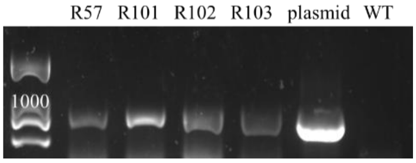

Figure S2. PCR check of *hyg* in different transformants and WT strain.

Table S1. List of genes that regulated by mating-type genes.

Perithecium development	Gene symbol	Accession number in PRJNA517412 (*M. sextelata*)	E value
Ascogonium (perithecium initials) formation	FGSG_00404*	SDUU01000001.1	2.56E-26
	FGSG_05239	SDUU01000019.1	3.97E-17
	FGSG_13708	SDUU01000008.1	3.51E-07
	FGSG_03916	SDUU01000002.1	1.12E-101
	FGSG_06039	SDUU01000036.1	0.00E+00
	FGSG_09896*	SDUU01000009.1	0.00E+00
Spore formation and and perithecium maturation	FGSG_00532	SDUU01000015.1	9.62E-19
	FGSG_09834*	SDUU01000015.1	9.12E-125
	FGSG_06059	SDUU01000002.1	9.64E-61

*Down regulated in overexpressing *mat1-2-1* in *Fusarium graminearum*

Table S2. Blast results of target genes of mating-type genes in *M. sextelata* and *M. importuna*.

Accession number in PRJNA517412 (*M. sextelata*)	JGI protein ID (*M. importuna*)	Query Cover	E value
SDUU01000001.1	JGI 372751	100%	0.0
SDUU01000019.1	JGI 484741	100%	0.0
SDUU01000008.1	JGI 504283	100%	0.0
SDUU01000002.1	JGI 481101	100%	0.0

SDUU01000036.1	JGI 533191	100%	0.0
SDUU01000009.1	JGI 479298	100%	0.0
SDUU01000015.1	JGI 543093	100%	0.0
SDUU01000015.1	JGI 533505	100%	0.0
SDUU01000002.1	JGI 517289	100%	0.0

All the ANOVA results showed in Table S3.1-10. ANOVA results of List VAR00001：1.00，2.00 and 3.00 represent M1 type, M2 type and Mix type, respectively.

Table S3. 1 Ratio of pileus to stipe.

ANOVA

VAR00002

	Sum of Squares	df	Mean Square	F	Sig.
Between Groups	.048	2	.024	1.036	.362
Within Groups	1.143	49	.023		
Total	1.192	51			

Multiple Comparisons

Dependent Variable:VAR00002

	(I) VAR00001	(J) VAR00001	Mean Difference (I-J)	Std. Error	Sig.	95% Confidence Interval Lower Bound	95% Confidence Interval Upper Bound
LSD	1.00	2.00	-.01660	.06306	.793	-.1433	.1101
		3.00	-.07285	.06306	.254	-.1996	.0539
	2.00	1.00	.01660	.06306	.793	-.1101	.1433
		3.00	-.05625	.04605	.228	-.1488	.0363
	3.00	1.00	.07285	.06306	.254	-.0539	.1996
		2.00	.05625	.04605	.228	-.0363	.1488

VAR00002

	VAR00001	N	Subset for alpha = 0.05 1
Duncan[a,,b]	1.00	8	1.1408
	2.00	22	1.1574
	3.00	22	1.2136
	Sig.		.242

Means for groups in homogeneous subsets are displayed.

a. Uses Harmonic Mean Sample Size = 13.895.

b. The group sizes are unequal. The harmonic mean of the group sizes is used. Type I error levels are not guaranteed.

Table S3. 2 Ratio of pileus length to width.

ANOVA

VAR00002

	Sum of Squares	df	Mean Square	F	Sig.
Between Groups	.088	2	.044	.938	.398
Within Groups	2.301	49	.047		
Total	2.389	51			

Multiple Comparisons

Dependent Variable:VAR00002

	(I) VAR00001	(J) VAR00001	Mean Difference (I-J)	Std. Error	Sig.	95% Confidence Interval	
						Lower Bound	Upper Bound
LSD	1.00	2.00	-.09950	.08946	.271	-.2793	.0803
		3.00	-.02436	.08946	.787	-.2041	.1554
	2.00	1.00	.09950	.08946	.271	-.0803	.2793
		3.00	.07514	.06533	.256	-.0562	.2064
	3.00	1.00	.02436	.08946	.787	-.1554	.2041
		2.00	-.07514	.06533	.256	-.2064	.0562

VAR00002

	VAR00001	N	Subset for alpha = 0.05
			1
Duncan[a,,b]	1.00	8	1.7279
	3.00	22	1.7522
	2.00	22	1.8274
	Sig.		.260

Means for groups in homogeneous subsets are displayed.

a. Uses Harmonic Mean Sample Size = 13.895.

b. The group sizes are unequal. The harmonic mean of the group sizes is used. Type I error levels are not guaranteed.

Table S3. 3 Size of hymenium.

ANOVA

VAR00002

	Sum of Squares	df	Mean Square	F	Sig.
Between Groups	.115	2	.058	3.462	.033
Within Groups	3.116	187	.017		
Total	3.231	189			

Multiple Comparisons

Dependent Variable:VAR00002

	(I) VAR00001	(J) VAR00001	Mean Difference (I-J)	Std. Error	Sig.	95% Confidence Interval	
						Lower Bound	Upper Bound
LSD	1.00	2.00	.05286*	.02187	.017	.0097	.0960
		3.00	.05046*	.02428	.039	.0026	.0984
	2.00	1.00	-.05286*	.02187	.017	-.0960	-.0097
		3.00	-.00240	.02357	.919	-.0489	.0441
	3.00	1.00	-.05046*	.02428	.039	-.0984	-.0026
		2.00	.00240	.02357	.919	-.0441	.0489

*. The mean difference is significant at the 0.05 level.

VAR00002

	VAR00001	N	Subset for alpha = 0.05	
			1	2
Duncan[a,,b]	2.00	75	.3596	
	3.00	50	.3620	
	1.00	65		.4125
	Sig.		.918	1.000

Means for groups in homogeneous subsets are displayed.

a.Uses Harmonic Mean Sample Size = 61.579.

b.The group sizes are unequal. The harmonic mean of the group sizes is used. Type I error levels are not guaranteed.

Table S3. 4 Size of excipulum.

ANOVA

VAR00002

	Sum of Squares	df	Mean Square	F	Sig.
Between Groups	.620	2	.310	31.129	.000
Within Groups	1.861	187	.010		
Total	2.481	189			

Multiple Comparisons

Dependent Variable:VAR00002

	(I) VAR00001	(J) VAR00001	Mean Difference (I-J)	Std. Error	Sig.	95% Confidence Interval	
						Lower Bound	Upper Bound
LSD	1.00	2.00	-.12252*	.01691	.000	-.1559	-.0892
		3.00	-.11692*	.01877	.000	-.1539	-.0799
	2.00	1.00	.12252*	.01691	.000	.0892	.1559

	3.00	.00560	.01821	.759	-.0303	.0415
3.00	1.00	.11692*	.01877	.000	.0799	.1539
	2.00	-.00560	.01821	.759	-.0415	.0303

*. The mean difference is significant at the 0.05 level.

VAR00002

	VAR00001	N	Subset for alpha = 0.05	
			1	2
Duncan[a,,b]	1.00	65	.1891	
	3.00	50		.3060
	2.00	75		.3116
	Sig.		1.000	.756

Means for groups in homogeneous subsets are displayed.

a. Uses Harmonic Mean Sample Size = 61.579.

b. The group sizes are unequal. The harmonic mean of the group sizes is used. Type I error levels are not guaranteed.

TableS3.5 Ratio of hymenium to excipulum.

ANOVA

VAR00002

	Sum of Squares	df	Mean Square	F	Sig.
Between Groups	57.598	2	28.799	33.803	.000
Within Groups	159.316	187	.852		
Total	216.914	189			

Multiple Comparisons

Dependent Variable:VAR00002

	(I) VAR00001	(J) VAR00001	Mean Difference (I-J)	Std. Error	Sig.	95% Confidence Interval Lower Bound	95% Confidence Interval Upper Bound
LSD	1.00	2.00	1.20630*	.15642	.000	.8977	1.5149
		3.00	1.07984*	.17363	.000	.7373	1.4224
	2.00	1.00	-1.20630*	.15642	.000	-1.5149	-.8977
		3.00	-.12646	.16852	.454	-.4589	.2060
	3.00	1.00	-1.07984*	.17363	.000	-1.4224	-.7373
		2.00	.12646	.16852	.454	-.2060	.4589

*. The mean difference is significant at the 0.05 level.

VAR00002

VAR00001		N	Subset for alpha = 0.05	
			1	2
Duncan[a,,b]	2.00	75	1.2687	
	3.00	50	1.3952	
	1.00	65		2.4750
	Sig.		.448	1.000

Means for groups in homogeneous subsets are displayed.

a. Uses Harmonic Mean Sample Size = 61.579.

b. The group sizes are unequal. The harmonic mean of the group sizes is used. Type I error levels are not guaranteed.

TableS3.6 Maximum size of the ascus**.**

ANOVA

VAR00002

	Sum of Squares	df	Mean Square	F	Sig.
Between Groups	161.007	2	80.503	12.184	.000
Within Groups	4287.983	649	6.607		
Total	4448.989	651			

Multiple Comparisons

Dependent Variable:VAR00002

	(I) VAR00001	(J) VAR00001	Mean Difference (I-J)	Std. Error	Sig.	95% Confidence Interval Lower Bound	95% Confidence Interval Upper Bound
LSD	1.00	2.00	-1.08195*	.27417	.000	-1.6203	-.5436
		3.00	-1.15841*	.24676	.000	-1.6430	-.6739
	2.00	1.00	1.08195*	.27417	.000	.5436	1.6203
		3.00	-.07646	.24151	.752	-.5507	.3978
	3.00	1.00	1.15841*	.24676	.000	.6739	1.6430
		2.00	.07646	.24151	.752	-.3978	.5507

*. The mean difference is significant at the 0.05 level.

VAR00002

VAR00001		N	Subset for alpha = 0.05	
			1	2
Duncan[a,,b]	1.00	170	19.3104	
	2.00	182		20.3923
	3.00	300		20.4688

	Sig.		1.000	.764

Means for groups in homogeneous subsets are displayed.

a. Uses Harmonic Mean Sample Size = 203.940.

b. The group sizes are unequal. The harmonic mean of the group sizes is used. Type I error levels are not guaranteed.

TableS3.7 Minimum size of the ascus.

ANOVA

VAR00002

	Sum of Squares	df	Mean Square	F	Sig.
Between Groups	206.876	2	103.438	19.455	.000
Within Groups	3450.529	649	5.317		
Total	3657.405	651			

Multiple Comparisons

Dependent Variable:VAR00002

	(I) VAR00001	(J) VAR00001	Mean Difference (I-J)	Std. Error	Sig.	95% Confidence Interval Lower Bound	95% Confidence Interval Upper Bound
LSD	1.00	2.00	-1.40278*	.24594	.000	-1.8857	-.9198
		3.00	-1.18288*	.22135	.000	-1.6175	-.7482
	2.00	1.00	1.40278*	.24594	.000	.9198	1.8857
		3.00	.21990	.21664	.310	-.2055	.6453
	3.00	1.00	1.18288*	.22135	.000	.7482	1.6175
		2.00	-.21990	.21664	.310	-.6453	.2055

*. The mean difference is significant at the 0.05 level.

VAR00002

	VAR00001	N	Subset for alpha = 0.05: 1	2
Duncan[a,,b]	1.00	170	16.1078	
	3.00	300		17.2907
	2.00	182		17.5106
	Sig.		1.000	.336

Means for groups in homogeneous subsets are displayed.

a. Uses Harmonic Mean Sample Size = 203.940.

b. The group sizes are unequal. The harmonic mean of the group sizes is used. Type I error levels are not guaranteed.

TableS3.8 The malformation degree of the ascus of different types.

ANOVA

VAR00002

	Sum of Squares	df	Mean Square	F	Sig.
Between Groups	.190	2	.095	8.698	.000
Within Groups	5.676	519	.011		
Total	5.866	521			

Multiple Comparisons

Dependent Variable:VAR00002

	(I) VAR00001	(J) VAR00001	Mean Difference (I-J)	Std. Error	Sig.	95% Confidence Interval	
						Lower Bound	Upper Bound
LSD	1.00	2.00	.04006*	.01115	.000	.0181	.0620
		3.00	.00000	.01134	1.000	-.0223	.0223
	2.00	1.00	-.04006*	.01115	.000	-.0620	-.0181
		3.00	-.04006*	.01115	.000	-.0620	-.0181
	3.00	1.00	.00000	.01134	1.000	-.0223	.0223
		2.00	.04006*	.01115	.000	.0181	.0620

*. The mean difference is significant at the 0.05 level.

VAR00002

	VAR00001	N	Subset for alpha = 0.05	
			1	2
Duncan[a,,b]	2.00	182	1.1677	
	1.00	170		1.2077
	3.00	170		1.2077
	Sig.		1.000	1.000

Means for groups in homogeneous subsets are displayed.

a.Uses Harmonic Mean Sample Size = 173.820.

b.The group sizes are unequal. The harmonic mean of the group sizes is used. Type I error levels are not guaranteed.

TableS3.9 Ascospore length.

ANOVA

VAR00002

	Sum of Squares	df	Mean Square	F	Sig.
Between Groups	977.710	2	488.855	56.608	.000
Within Groups	1787.613	207	8.636		
Total	2765.323	209			

Multiple Comparisons

Dependent Variable:VAR00002

	(I) VAR00001	(J) VAR00001	Mean Difference (I-J)	Std. Error	Sig.	95% Confidence Interval	
						Lower Bound	Upper Bound
LSD	1.00	2.00	-2.92900*	.49673	.000	-3.9083	-1.9497
		3.00	-5.27457*	.49673	.000	-6.2539	-4.2953
	2.00	1.00	2.92900*	.49673	.000	1.9497	3.9083
		3.00	-2.34557*	.49673	.000	-3.3249	-1.3663
	3.00	1.00	5.27457*	.49673	.000	4.2953	6.2539
		2.00	2.34557*	.49673	.000	1.3663	3.3249

*. The mean difference is significant at the 0.05 level.

VAR00002

	VAR00001	N	Subset for alpha = 0.05		
			1	2	3
Duncan[a]	1.00	70	16.4291		
	2.00	70		19.3581	
	3.00	70			21.7037
	Sig.		1.000	1.000	1.000

Means for groups in homogeneous subsets are displayed.

a. Uses Harmonic Mean Sample Size = 70.000.

TableS3.10 Ascospore width.

ANOVA

VAR00003

	Sum of Squares	df	Mean Square	F	Sig.
Between Groups	485.737	2	242.868	76.693	.000
Within Groups	655.516	207	3.167		
Total	1141.253	209			

Multiple Comparisons

Dependent Variable:VAR00003

	(I) VAR00001	(J) VAR00001	Mean Difference (I-J)	Std. Error	Sig.	95% Confidence Interval	
						Lower Bound	Upper Bound
LSD	1.00	2.00	-1.62071*	.30080	.000	-2.2137	-1.0277
		3.00	-3.71529*	.30080	.000	-4.3083	-3.1223
	2.00	1.00	1.62071*	.30080	.000	1.0277	2.2137
		3.00	-2.09457*	.30080	.000	-2.6876	-1.5016

3.00	1.00	3.71529*	.30080	.000	3.1223	4.3083
	2.00	2.09457*	.30080	.000	1.5016	2.6876

*. The mean difference is significant at the 0.05 level.

VAR00003

	VAR00001	N	Subset for alpha = 0.05		
			1	2	3
Duncan[a]	1.00	70	9.2853		
	2.00	70		10.9060	
	3.00	70			13.0006
	Sig.		1.000	1.000	1.000

Means for groups in homogeneous subsets are displayed.

a. Uses Harmonic Mean Sample Size = 70.000.

Table S4. 1 The transcription level of mating-type genes in pileus.

	**M1 type**	**M2 type**	**Mix**
mat1-1-1	0.552±0.015	0.766±0.016	0.342±0.011
mat1-1-10	0.49±0.022	0.559±0.021	0.509±0.019
mat1-1-11	0.355±0.006	0.484±0.008	0.196±0.007
mat1-2-1	0.387±0.017	0.462±0.031	0.663±0.028

Table S4. 2 The transcription level of ascus development-related genes in pileus.

	**M1 type**	**M2 type**	**Mix**
JGI372751	0.817±0.015	0.625±0.01	0.24±0.01
JGI484741	0.919±0.021	0.954±0.026	0.952±0.023
JGI504283	0.55±0.009	0.656±0.009	0.618±0.014
JGI481101	0.667±0.018	0.559±0.015	0.595±0.021
JGI533191	0.578±0.025	0.71±0.026	0.685±0.025
JGI479298	0.723±0.02	0.636±0.008	0.443±0.013
JGI543093	0.887±0.004	0.918±0.009	0.91±0.011
JGI533505	0.893±0.014	0.375±0.006	0.159±0.006
JGI517289	0.535±0.008	0.559±0.015	0.279±0.006
